# Supplementary material for: Captopril, a Renin–Angiotensin System Inhibitor, Attenuates Tumour Progression in the Regenerating Liver Following Partial Hepatectomy
Source: Int J Mol Sci. 2022 May 9;23(9):5281. doi: 10.3390/ijms23095281 (PMC9105412; doi:10.3390/ijms23095281)
Supplement: Supplementary file 1 [file ijms-23-05281-s001.zip › ijms-1692385-supplementary.pdf]

Supplementary Materials

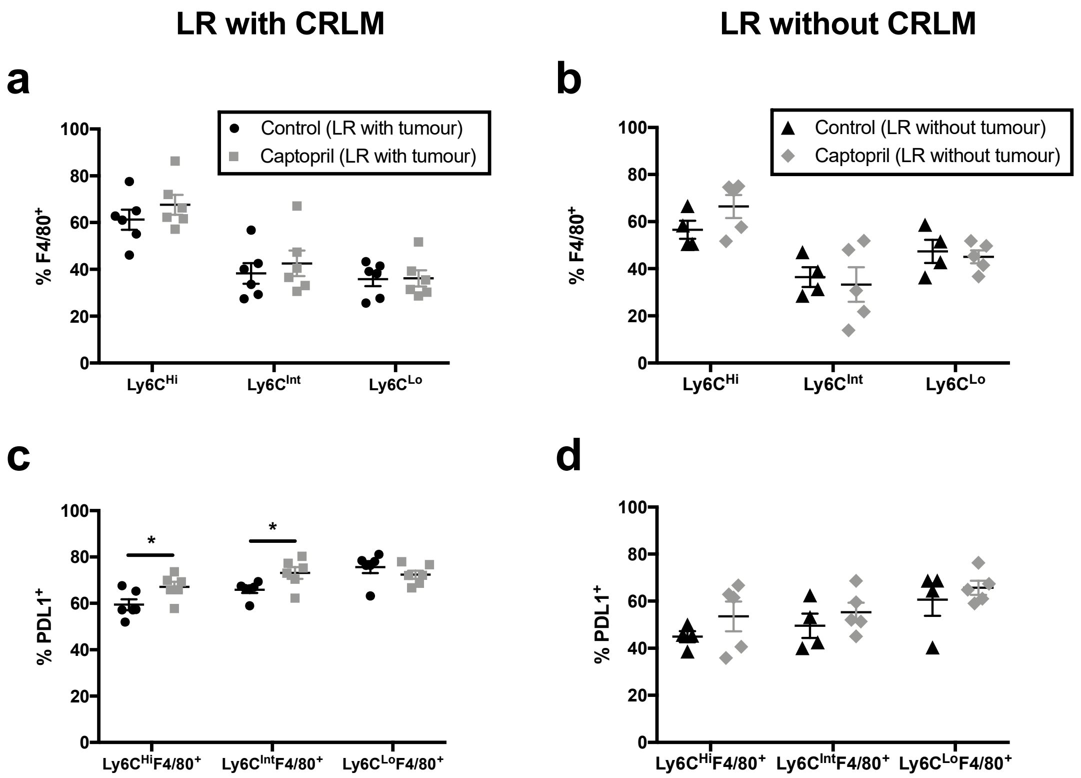

Figure S1. MDSC subpopulation analysis in mice with CRLM and without CRLM. One group of mice underwent surgical induction of colorectal liver metastasis via splenic injection (Day 0), and one group of mice did not undergo tumour induction. From Day 4 to Day 16, intraperitoneal injections of captopril 250mg/kg/day or saline (control) were administered to all mice in both groups. On Day 7, all mice underwent a 70% partial hepatectomy. All mice were culled on Day 16, and liver and tumour specimens were retrieved for flow cytometry: (**a-b**) proportion of each MDSC subset expressing F4/80 in liver with CRLM (**a**) and without CRLM (**b**); (**c-d**) proportion of each F4/80+ MDSC subset expressing PDL1 in liver with CRLM (**c**) and without CRLM (**d**). .

Table S1. Ratios of total $CD4^{+}/CD8^{+}$, $CD8^{+}$/DN T and $CD4^{+}$/DN T with their respective ratios for $CD44^{+}CD69^{+}$ and $CD44^{+}CD69^{+}$PD-1 populations. Data for liver and tumour are shown. Mice underwent colorectal liver metastasis tumour induction and four days later commenced treatment with either control saline or RASi captopril (250mg/kg/day via intraperitoneal injection). One week following tumour induction, 70% hepatectomy was performed. Mice were culled on Day 16, and liver and tumour specimens obtained for flow cytometry. Significant differences were determined by an unpaired, two-tailed Student's *t*-test. Significant p values are in bold. SD–standard deviation.

	**LIVER: TOTAL**			**LIVER: $CD44^{+}CD69^{+}$**			**LIVER: $CD44^{+}CD69^{+}PD\text{-}1^{+}$**			**TUMOR: TOTAL**			**TUMOR: $CD44^{+}CD69^{+}$**			**TUMOR: $CD44^{+}CD69^{+}PD\text{-}1^{+}$**		
**RATIOS**	CD4/ CD8	CD8/ DN T	CD4/ DN T	CD4/ CD8	CD8/ DN T	CD4/ DN T	CD4/ CD8	CD8/ DN T	CD4/ DN T	CD4/ CD8	CD8/ DN T	CD4/ DN T	CD4/ CD8	CD8/ DN T	CD4/ DN T	CD4/ CD8	CD8/ DN T	CD4/ DN T
**CAPTOPRIL (MEAN)**	0.91	1.13	0.87	0.45	1.63	0.65	0.44	2.03	0.77	6.09	0.38	2.05	5.06	0.72	3.54	5.71	0.79	4.35
**SD**	0.36	0.71	0.25	0.25	0.61	0.26	0.27	0.79	0.36	3.17	0.12	0.33	1.77	0.08	0.91	1.95	0.10	0.97
**SALINE (MEAN)**	1.57	1.10	1.63	1.00	1.71	1.54	1.33	2.58	3.16	7.46	0.38	2.23	8.89	0.61	4.05	11.19	0.72	6.09
**SD**	0.35	0.40	0.28	0.33	1.26	0.91	0.31	1.86	1.74	4.91	0.18	0.35	7.09	0.28	0.83	8.88	0.36	1.46
**P VALUE**	**0.01**	0.93	**0.001**	**0.01**	0.90	0.06	**0.0007**	0.55	**0.01**	0.60	0.98	0.39	0.23	0.42	0.35	0.21	0.71	**0.04**

Table S2. Ratios of total $CD4^+/CD8^+$, $CD8^+$/DN T and $CD4^+$/DN T with their respective ratios for $CD44^+CD69^+$ and $CD44^+CD69^+$PD-1 populations. Mice commenced treatment with either control saline or RASi captopril 250mg/kg/day and did not undergo tumour induction. Briefly, 70% hepatectomy was performed 3 days after the commencement of treatment or control, and mice were culled nine days following partial hepatectomy in keeping with prior experiments. Liver specimens were retrieved for flow cytometry. Significant differences were determined by an unpaired, two-tailed Student's *t*-test. Significant p values are in bold. SD–standard deviation.

	**LIVER: TOTAL**			**LIVER: $CD44^+CD69^+$**			**LIVER: $CD44^+CD69^+PD\text{-}1^+$**		
**RATIOS**	CD4/ CD8	CD8/ DN T	CD4/ DN T	CD4/ CD8	CD8/ DN T	CD4/ DN T	CD4/ CD8	CD8/ DN T	CD4/ DN T
**CAPTOPRIL (MEAN)**	0.29	7.48	2.25	0.13	8.44	1.15	0.10	17.41	1.97
**SD**	0.10	2.29	1.20	0.05	2.53	0.62	0.04	9.50	1.71
**SALINE (MEAN)**	0.86	2.29	1.90	0.47	1.76	0.82	0.55	3.15	1.73
**SD**	0.15	0.48	0.16	0.13	0.67	0.34	0.16	0.38	0.48
**P VALUE**	**0.0001**	**0.001**	0.54	**0.0006**	**0.0005**	0.33	**0.0002**	**0.01**	0.77
